# Disparity in hospital beds’ allocation at the county level in China: an analysis based on a Health Resource Density Index (HRDI) model

**DOI:** 10.1186/s12913-023-10266-4

**Published:** 2023-11-23

**Authors:** Zuobao Wang, Lin Dong, XinYi Xing, Zhe Liu, Yuxiang Zhou

**Affiliations:** School of Humanities and Law, Northeastern University, 195 Chuangxin Road, Hunnan District, Shenyang, 110169 Liaoning Province China

**Keywords:** Hospital beds, Health resource density index, Disparity, County, China

## Abstract

**Background:**

As approximately 3/4 of the population lives in county-level divisions in China, the allocation of health resources at the county level will affect the realization of health equity. This study aims to evaluate the disparity in hospital beds at the county level in China, analyze its causes, and discuss measures to optimize the allocation.

**Methods:**

Data were drawn from the Chinese County/City Statistical Yearbook (2001–2020). The health resource density index (HRDI) was applied to mediate between the influence of demographic and geographical factors on the allocation of hospital beds. The trends of HRDI allocation were evaluated through the growth incidence curve and the probability density function. The regional disparity in the HRDI was examined through the Lorenz curve, and Dagum Gini coefficient. The contribution of the Gini coefficient and its change were assessed by using the Dagum Gini decomposition method.

**Results:**

From 2000 to 2019, the number of hospital beds per thousand people at the county level in China increased dramatically by 1.49 times. From the aspect of the HRDI, there were large regional disparities at the national level, with a Gini coefficient of 0.367 in 2019 and in the three subregions. In 2019, the Gini coefficient of the HRDI exhibited regional variations, with the highest value observed in the western region, followed by the central region and the eastern region. Decomposition reveals that the contribution of interregional disparity changed from the dominant factor to the least important factor, accounting for 29.79% of the overall disparity and the contribution of trans-variation intensity increased from 29.19% to 39.75%, whereas the intraregional disparity remained stable at approximately 31% and became the second most important factor.

**Conclusion:**

The regional disparity in hospital beds allocation at the county level in China was large and has not improved substantially. Trans-variation intensity was the main reason for the overall disparity and changes, and the intraregional disparity was more important than the interregional disparity for the overall disparity.

## Background

Health equity is an important embodiment of social equity. In 1978, the Alma-Ata Declaration reaffirmed that health is a fundamental human right, and the attainment of the highest possible level of health is the most important worldwide social goal. Since then, health equity has become an important issue in public health. In 2000, the World Health Organization (WHO) defined health equity such that whether it is defined in social, economic, demographic, or geographical terms, there is no health gap [1].

As health status is the direct output of health services, to some extent, the degree of health equity is determined by whether people can access equitable healthcare facilities and services [2–4], which has been defined as a fundamental human right, and is the key for people to enjoy other human rights [5]. Therefore, several scholars have investigated the availability of healthcare resources as one of the main criteria for measuring the equity within the health system, and the inequitable distribution of healthcare services is recognized as a major barrier to advancing health equity [6]. From the perspective of the distribution of health resources, the WHO provided another definition of “health equity” as the fair distribution of resources needed for health, fair access to opportunities for wellness, and fairness in the support offered to people when ill [7].

AS the largest developing country in the world, China has enjoyed great economic development success since the implementation of the Reform and Opening-up policy in 1978. Along with economic development, China has paid great attention to and prioritized resources for social development, leading to significant improvements in access to healthcare, education, and other public services. In terms of healthcare, by the end of 2020, in China, licensed doctors (assistants) per thousand people reached 2.9, compared with 1.7 in 2000. The number of registered nurses per thousand people was 3.34, compared with 1 in 2000. The number of hospital beds per thousand people reached 6.46, more than twice the number in 2002 (2.49). These remarkable improvements have greatly enhanced the health status of Chinese citizens, with a life expectancy of 77.3 years in 2019, up 10.9 years from 1979, which is now among the top in developing countries [8].

Despite these evident advances, China’s healthcare system still faces some substantial challenges, one of which is the unequal distribution of health resources. In China, publicly owned hospitals have long been dominant and provide a considerable share of health services [9]. However, in reality, there are large rural–urban, interregional, and intraregional gaps in China, and due to these gaps, fiscal disequilibrium and regional disparities in public investment have prevailed [10]. Thus, an uneven distribution of public services, including education, social security, cultural activities, sports, and healthcare, across regions inevitably occurs [11–13].

In terms of health resources, a number of studies have shown population- or geography-based disparities in China in the health workforce, health services, and facilities including institutions, hospital beds, operating rooms, and medical equipment [14–16]. The number of hospital beds plays an important role in determining healthcare capacity. Undoubtedly, increasing the supply of hospital beds will improve health services, such as hospitalization and care provision [17–19]. Especially when facing a disaster, the availability of empty beds that could receive patients is a fundamental component of hospitals’ surge capacity [20–22]. For example, during the pandemic of COVID-19, as most hospital beds were occupied by COVID-19 patients, there arose an acute crisis of beds and even disruptions in routine health service provision [23–25].

According to the WHO, China has enough hospital beds to accommodate only 0.4% of its population under normal operating circumstances [26]. Thus, to provide equal basic healthcare services and increase the surge capacity for disaster response, an equal distribution of hospital beds is needed. However, in China, hospital beds tend to be distributed in wealthier areas and cities, causing a remarkably large inequality in hospital beds according to previous studies [27–29], and this inequality will further exacerbate the disparities in surge capacity between regions during disasters and lead to higher inequality in people's health status.

Despite the importance of the equal distribution of hospital beds, there still exist some research gaps in the literature, which has mainly analyzed the allocation of health resources, including hospital beds, in one province or one region [30, 31] or focuses on the whole country at the provincial level or prefecture level [15, 28, 32, 33]. For example, Yang et al. assessed the equality of the distribution of hospital beds among provinces in China during the 1998–2016 period and found that the Gini coefficient of hospital beds decreased from 2004 until 2013 and then rose to bounce back [34]. Dai et al. measured the discrepancies in medical services including hospital beds among 16 cities and prefectures in Yunnan Province in China from 2009 to 2013, finding an obvious downward trends in the Theil index of hospital beds [35]. Clearly, if the intraregional disparity (at the provincial level or prefecture level) is ignored, the level of inequality will be underestimated. In particular, there are 2860 county-level divisions in China, among which there are approximately 2000 rural counties, and approximately 3/4 of the population lives there. The allocation of hospital beds in counties will certainly affect the health services that most people can access and, thus, dominate the overall inequality of health resources and people’s health status in China.

Although a few articles have studied the allocation of health resources at the county level, most of them compared levels of hospital beds’ allocation [36] without calculating an inequality index, or estimated the inequality based on only population or geography. However, due to the differences in population and geography between regions, the results are always quite different or even opposite. For instance, Li et al. found that the equality of traditional Chinese medicine resources by population (Gini coefficient ranging from 0.1 to 0.3) was better than that by geographical region (Gini coefficient > 0.5) [37]. Yao [38] and Lu [39] concluded that the gap in the allocation of public health institutions per square kilometer was larger than that per 10,000 people. Thus, in this paper, we fill this research gap by estimating the disparity in the number of hospital beds per thousand people at the county level in China, using the Health Resource Density Index (HRDI) based on the aspects of both population and geography.

As will be seen, this research makes four contributions: (1) We focus on the allocation of hospital beds at the county level, improving upon the limited existing studies, which lack adequate attention to county-level health resource divisions. (2) We calculate the Health Resource Density Index (HRDI) to mediate between the influence of demographic and geographical factors on the allocation of hospital beds, avoiding the biases caused by a single population or geographical perspective. (3) We estimate the disparity by calculating the Dagum Gini coefficient and decompose it, seeking to pinpoint the source of the disparity. (4) The findings of this paper have implications for designing policies to promote the equal distribution of hospital beds and other health resources at the county level in China.

The rest of this paper is structured as follows. Data and methods section introduces the data and methods used. Results section presents the results of the measurements of the HRDI of hospital beds, the growth pattern, the disparity, and the decomposition; Discussion section  discusses the results, focusing on the contributions, policy implications, and limitations of this study; and Conclusions section draws the conclusions.

## Data and methods

### Data sources

In this paper, we used data on hospital beds, population, and area at the county level obtained from China County/City Statistical Yearbook, 2001–2020, released by the National Bureau of Statistics of China.

According to the “Statistical Report of Administrative Divisions in China in 2000” released by the Ministry of Civil Affairs, there were 2860 county level administrative units, including 2052 counties (including counties, county-level cities and autonomous counties) in rural areas and 808 municipal districts in urban areas, except for the Hong Kong Special Administrative Region (SAR), Macao SAR, and Taiwan province.

Due to the weaker autonomy in administrative power, fiscal expenditure, and public service provision compared to counties, municipal districts have a comparatively strong dependence on cities [40]. Additionally, counties have larger geographical areas and are situated at a greater distance from other regions, resulting in hospital bed resources primarily serving the residents within their own county. In contrast, municipal districts are closely interconnected, and residents in these areas may seek medical services outside their own district. Applying the same methodology used for counties and county-level cities to municipal districts may lead to an overestimation of hospital bed levels. Therefore, this study defines the research objects as counties (including counties, county-level cities and autonomous counties) while excluding municipal districts.

To make a longitudinal comparison, this paper incorporated all counties in 2000 even though some of these counties may have been reclassified as municipal districts in the adjustment of administrative divisions. Owing to data availability, the number of counties for which we obtained the number of hospital beds ranged from 2027 to 2082 during the 2000–2019 period, where there were 0.93–1.03 billion people, constituting a proportion of 72.0%–74.4% of the total population of China (Table 1).

**Table 1:** The number of sample counties with population numbers

Year	Number of sample counties	Population living in the case counties (ten thousands)	Proportion in the total population (%)
2000	2,043	93,023	73.39
2001	2,042	93,268	73.08
2002	2,042	93,636	72.89
2003	2,047	93,969	72.72
2004	2,057	94,442	72.65
2005	2,057	94,826	72.52
2006	2,027	94,653	72.01
2007	2,054	96,804	73.26
2008	2,058	97,313	73.28
2009	2,055	97,914	73.37
2010	2,058	99,642	74.31
2011	2,064	100,408	74.42
2012	2,070	100,945	74.27
2013	2,069	100,949	73.83
2014	2,069	101,615	73.82
2015	2,072	101,855	73.63
2016	2,075	102,436	73.57
2017	2,075	102,630	73.30
2018	2,077	102,756	73.11
2019	2,082	103,008	73.05

### Measuring tools

This study began by calculating the health resource density index (HRDI) for hospital beds within each county. Subsequently, growth incidence curves were constructed to provide a visual representation of the growth patterns of counties at various percentiles. To gain a more comprehensive understanding of the distribution and variation in hospital beds among counties, kernel density estimation was employed. Finally, we estimated the Dagum Gini coefficient and conducted a decomposition analysis to quantitatively evaluate the level of and changes in regional disparities in hospital beds across counties over time, and to identify the contributing factors.

### Health resource density index

The health resource density index (HRDI) proposed by Zheng and Ling [41], was applied to evaluate the allocation of hospital beds in a certain county from the perspective of both population and geography. The formula of the HRDI is as follows:

1 $$HRDI= \sqrt{\left(r/p\right) \times \left(r/a\right)}$$

where *r* is the number of hospital beds, *p* is the registered population in thousands due to the unavailability of resident population data for all counties for each year from 2000 to 2020, and *a* is the area in 100 square kilometers. It is important to acknowledge that in certain counties, particularly those located on the west side of the Hu Huanyong line, where numerous areas remain inaccessible, using the entire geographical area for calculations may lead to an underestimation of the level of hospital beds. For a more precise assessment, the ideal approach would entail utilizing data on built-up areas or places with frequent human mobility. Regrettably, the available data cover only built-up areas within county towns or city districts. Consequently, we are obliged to employ the entire geographical area as an alternative dataset when calculating the HRDI.

### Growth incidence curve

We present the growth incidence curve (GIC) proposed by Ravallion and Chen [42] to show how the growth rate of the *p*th percentile varies across counties in ascending order of HRDI. The growth rate of the HRDI of the *p*th percentile from time *t*1 to *t*2 can be calculated as follows (it traces out what Ravallion and Chen [42] termed the GIC):

2 $$g_{t1-t2}\left(p\right)\;=\;\frac{y_{t2\;\left(p\right)}}{y_{t1\;\left(p\right)}}\;-1=\;\frac{{ L'}_{ t\mathit{2}}\;\left(p\right)}{{L'}_{t\mathit{1}}\;\left(p\right)}\;\left(Y_{t1-t2}\;+1\right)-1$$

where $y_t\;\left(p\right)$  is the HRDI of the *p *th percentile at time *t*; $L_t\left(p\right)$  is the Lorenz curve and  $L{'}_t\left(p\right)$ is the slope of the curve; and  $Y_{t1-t2}$ is the growth rate of the mean HRDI for all counties from time *t*1 to *t*2. The distribution becomes more unequal if the GIC is upward sloping, whereas if the GIC is downward sloping the distribution becomes more equal. When inequality does not change, the curve is a flat line [42].

### Probability density function

The probability density function is used to describe the continuous probability distribution of the HRDI at the county level through Gaussian kernel density estimation [43] as follows:

3 $$f \left(x\right)= \frac{1}{nh} \sum_{i=1}^{n}K\frac{{x}_{1}- \overline{x}}{h }$$

where *x*_*i*_ is the observed data point, $\overline x$ is the mean,  *h* is a bandwidth that acts as a ’smoothing ‘ parameter, *n* is the number of data points (i.e., the number of counties), and *K* is called the kernel function. This article uses the Gaussian kernel, which is one of the most popular choices.

### Dagum Gini coefficient

After graphically presenting the inequality using the Lorenz curve, the paper applies the Dagum Gini index to calculate inequality. The Gini coefficient is the most commonly used indicator of inequality and has been promoted as a measure for health inequality [15, 44]. Compared with the traditional Gini coefficient, the Dagum Gini coefficient and its decomposition method consider the subsample distribution state; it can effectively solve the problem of the crossover phenomenon between samples and, as a result, make more accurate conclusions on decomposing the sources of disparity. Therefore, this paper adopts the Dagum Gini coefficient and its decomposition method to evaluate the disparity and its sources. Referring to Dagum [45], the Dagum Gini coefficient can be defined as follows:

4 $$G=\; \frac{{\sum\limits_{j=1}^k}\; {\sum\limits_{h=1}^{k}}\;{\sum\limits_{i=1}^{n_j}}\; {\sum\limits_{r=1}^{n_h}}\; \left|x_{ji}\; -x_{hr}\right|}{2n^2\mu}$$

where *G* represents the Dagum Gini coefficient, *k* represents the number of subregions in the sample (in this paper, the counties are roughly grouped into three subregions, namely the eastern, central, and western regions, thus *k* equals 3), *n* represents the number of total counties, *x*_*ji*_ and *x*_*hr*_ represent the HRDI of county *i* in subregion *j* and county *r* in subregion *h*, respectively, and *n*_*j*_ and *n*_*h*_ represent the number of counties in subregions *j *and *h*, respectively. The total Dagum Gini coefficient can be decomposed into three parts: intraregional differences (*G*_*w*_), interregional differences (*G*_*nb*_) , and the intensity of the trans-variation between regions (*G*_*t*_).

## Results

### Levels and trends of the allocation of hospital beds

Table 2 shows the levels and trends of hospital beds in counties in China and the three subregions. Between 2000 and 2019, the number of hospital beds per thousand people in all counties increased by 1.49 times, rising from 1.93 to 4.80. However, this figure remained lower than the national average, including counties and cities in 2019 (6.3 beds per 1,000 people). The most significant increase occurred in western China, where the number of hospital beds per thousand people surged by 158.11%. This growth further widened the gap between western China and the other two subregions. Despite the majority of county-level hospital beds being situated in eastern China, the number of hospital beds per thousand people in this region was smaller than that of the western region in each year and often smaller than that of the central region, primarily due to its larger population size. Furthermore, the gap between western China and the other two subregions expanded as a result of its more substantial growth.

**Table 2:** Levels and trends of hospital beds at the county level in China, 2000–2019

Year	Hospital beds per thousand people				Hospital beds per 100 square kilometers				HRDI for hospital beds			
	**Total**	**East**	**Central**	**West**	**Total**	**East**	**Central**	**West**	**Total**	**East**	**Central**	**West**
2000	1.93	1.83	1.90	2.01	50.32	79.61	53.07	30.42	8.32	11.35	9.16	5.88
2001	1.95	1.84	1.91	2.04	51.15	80.72	53.32	31.32	8.42	11.48	9.19	6.00
2002	1.95	1.84	1.92	2.04	51.28	82.37	53.29	30.95	8.49	11.63	9.18	6.11
2003	1.95	1.86	1.88	2.05	51.43	83.67	52.61	30.87	8.48	11.77	9.02	6.10
2004	1.97	1.88	1.90	2.06	52.13	85.59	54.06	30.35	8.59	11.97	9.17	6.13
2005	2.01	1.96	1.91	2.10	53.37	90.62	54.43	29.92	8.82	12.60	9.23	6.23
2006	2.08	2.04	1.95	2.19	56.66	96.05	57.51	31.67	9.24	13.25	9.60	6.50
2007	2.18	2.13	2.05	2.30	59.74	101.33	60.70	33.86	9.71	13.89	10.08	6.93
2008	2.30	2.27	2.16	2.41	64.85	109.48	65.53	37.11	10.47	14.92	10.84	7.49
2009	2.46	2.40	2.29	2.60	69.69	117.02	69.38	40.91	11.20	15.85	11.44	8.20
2010	2.62	2.58	2.42	2.78	76.30	127.09	75.03	45.54	12.13	17.13	12.28	8.92
2011	2.80	2.76	2.61	2.96	81.81	136.85	81.71	47.91	13.04	18.38	13.29	9.59
2012	3.10	3.00	2.87	3.32	91.69	149.32	90.92	56.62	14.52	20.02	14.73	10.97
2013	3.41	3.28	3.22	3.62	102.88	164.46	103.38	64.51	16.21	21.98	16.67	12.33
2014	3.68	3.43	3.50	3.97	111.19	172.03	115.06	70.95	17.47	23.00	18.31	13.48
2015	3.93	3.68	3.74	4.21	119.12	183.14	125.25	75.53	18.69	24.46	19.82	14.36
2016	4.12	3.83	3.89	4.45	125.36	194.64	129.49	79.85	19.66	25.82	20.59	15.22
2017	4.35	4.11	4.15	4.63	134.31	208.60	139.68	84.88	20.97	27.68	22.06	16.09
2018	4.57	4.27	4.38	4.88	141.22	217.61	148.30	89.31	22.00	28.82	23.35	16.89
2019	4.80	4.45	4.54	5.20	148.94	228.79	154.61	95.72	23.15	30.14	24.35	18.00
Growth rate (%)	148.69	143.10	138.94	158.11	195.98	187.39	191.36	214.63	178.22	165.55	165.87	206.13

In terms of hospital beds per 100 square kilometers, the increase rates of the whole country and the three subregions are all higher than the growth rate based on population. Western counties exhibited the highest growth rate of 214.63%, while the eastern region had the lowest growth rate of 187.39%. Nevertheless, due to its expansive geographical area, the average number of hospital beds per 100 square kilometers in western counties remained significantly lower than that in eastern counties.

**Fig. 1:**
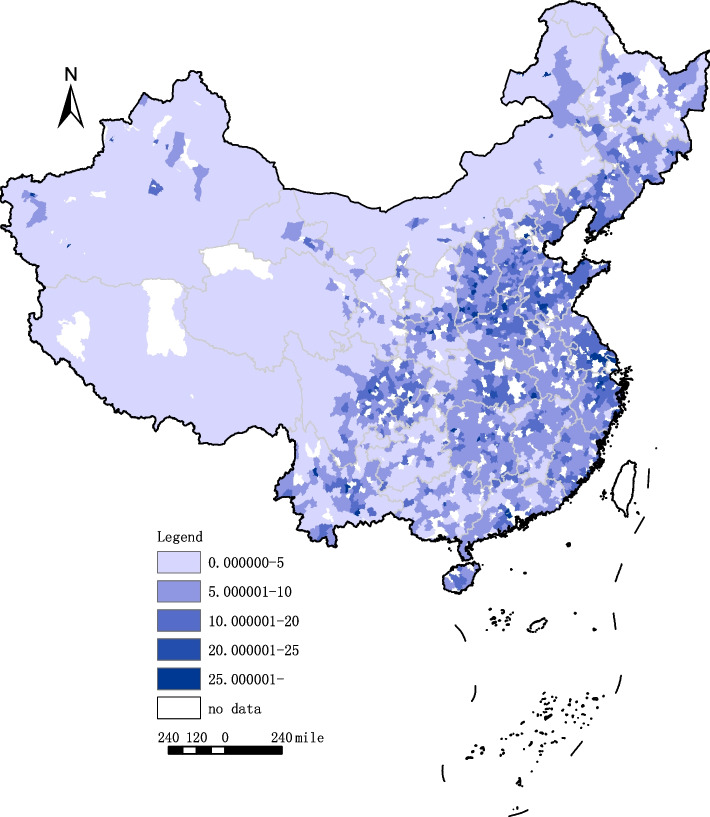
Geographical distribution of the HRDI of counties in China, 2000

**Fig. 2:**
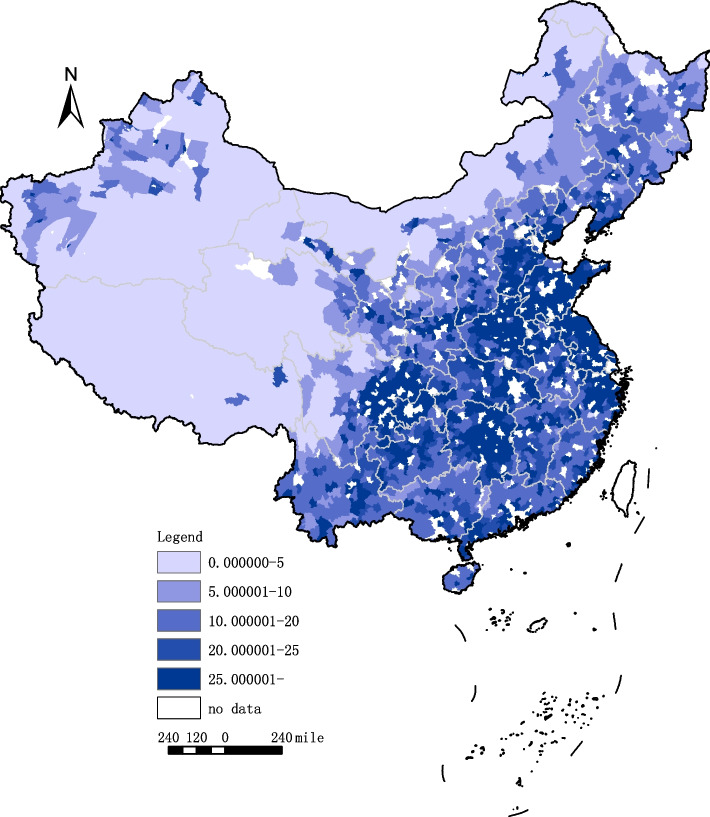
Geographical distribution of the HRDI of counties in China, 2019

With respect to the HRDI, in each year, the mean value of eastern counties was higher than that of central counties, which was also higher than that of western counties. To intuitively show the changes in the HRDI of all counties in the geographical and temporal dimensions from 2000 to 2019, this paper made a geographical distribution map (Figs. 1 and 2), using a color scale to indicate the HRDI level. The darker the blue is, the higher the level of the HRDI. According to Figs. 1 and 2, the counties with relatively high HRDIs are mainly concentrated on the east side of the “Huhuanyong Line”, i.e., eastern and central China. In addition, compared to 2000, the area with a darker color expanded in 2019, indicating that the HRDI of counties in China increased during this period.

From 2000 to 2019, the mean HRDI of all counties increased from 8.32 to 23.15 with a growth rate of 178.22%. Notably, western counties experienced the most substantial increase at 206.13%, which was approximately 40 percentage points higher than that observed in eastern and central counties. Given that the growth rate of the HRDI was higher in counties with lower initial levels (western counties) than in counties with higher initial levels, it is reasonable to conclude that the disparity in the HRDI may have improved. In the following section, we will analyze the disparity in hospital beds at the county level, focusing solely on the HRDI.

**Fig. 3:**
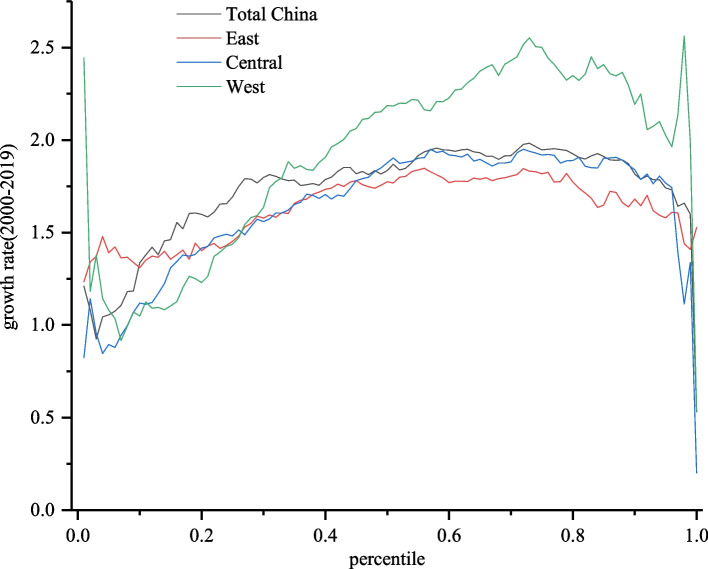
Growth incidence curves of the HRDI of counties in China from 2000 to 2019. Note: The figure was generated using Origin software based on the data calculated using Eq. (2)

Figure 3 shows the growth incidence curves of the HRDI of the whole country and three subregions, which report the same findings as Table 2 when comparing the growth rates of the three subregions. It can be observed in Fig. 3 that from the 25th percentile to the end of the distribution, the GIC for the western region was above that of the central region, which was also above the curve of the eastern region. Moreover, the distance between the GICs of the western and central regions was larger than that between the central and eastern regions. The distance was so large for most counties that although in the bottom part of the distribution the GIC for the west was below the curves for the other two subregions. We also drew the same conclusion as in Table 2, that is, the HRDI of western counties increased more than that of the central and eastern regions. From the 20th percentile, the curve of the central region was above the GIC of the eastern region, but the gap was small, and at the bottom part, the position of these two curves reversed. Thus, the growth rates of the HRDI for the central and eastern regions may be close to each other, which is also consistent with the conclusion derived from Table 2.

At the national level, the GIC showed a negative slope for the lowest three percent of counties and then increased steeply until the 30th percentile. Between the 30th and 80th percentiles, the curve fluctuated and then decreased.

Comparing the curves for the three subregions, the GIC for the western region exhibited the highest degree of fluctuation, whereas the curve for the eastern region displayed the least variation. Specifically, in the western region, between the 1st and 8th percentiles, between the 73rd and 96th percentiles, and between the 98th and 100th percentiles, the GIC had a negative slope, while it was positive in other parts. In the central region, the GIC had a negative slope between the 1st and 7th percentiles, after which it displayed an upward shift until the 87th percentile. In contrast, the GIC of the eastern region fluctuated smoothly.

From the shape of the GICs of these three subregions, we can conclude that in the western and central regions, the growth rate of counties with a high HRDI was higher than that of counties with a lower HRDI. Thus, clearly, the disparity in the HRDI between counties in the western and central regions worsened, and the changes in the western region were larger than those in the central region. However, as the GIC in the eastern region and at the national level fluctuated without drastic change, it is not easy to draw accurate conclusions about how the disparity in the HRDI between counties changed in the eastern region and in the whole country.

**Fig. 4:**
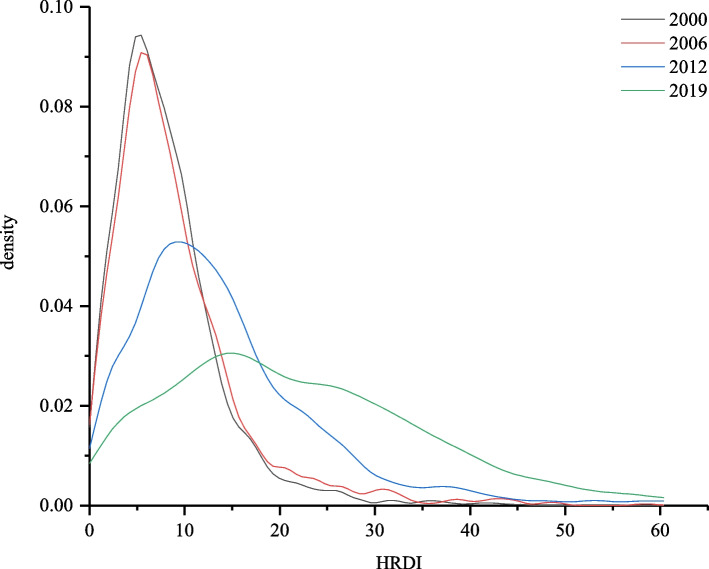
Probability density function of the HRDI allocation in counties in China in 2000, 2006, 2012, and 2019. Note: The figure was generated using Origin software based on the data calculated using Eq. (3)

Directly linked to the differences in growth rates across quantiles, the allocation of the HRDI among counties changed as shown by the probability density function curves (PDF) curves in Fig. 4. It is evident that the curves for 2000 and 2006, both displaying a more pronounced right tail, closely overlapped. This finding suggests that between 2000 and 2006, there was a prevalence of counties in the lower segment of the distribution, and the value of and disparity in the HRDI within counties remained relatively stable. In fact, according to Table 1, at the national level from 2000 to 2006, the mean HRDI increased by only 0.92, with a rate of 11.06%.

During the 2006–2012 and 2012–2019 periods, it is clear that the PDF curves shifted rightward and became wider and shallower, indicating that the mean value of the HRDI became larger, and the distribution became more dispersed. In addition, we can see that the left tail of the curves became thinner, while the right tail became thicker from 2006 to 2019. This finding implies that during this period, the proportion of counties with a lower HRDI declined and those with a higher HRDI increased.

## Description and decomposition of the regional disparity in hospital beds

**Fig. 5:**
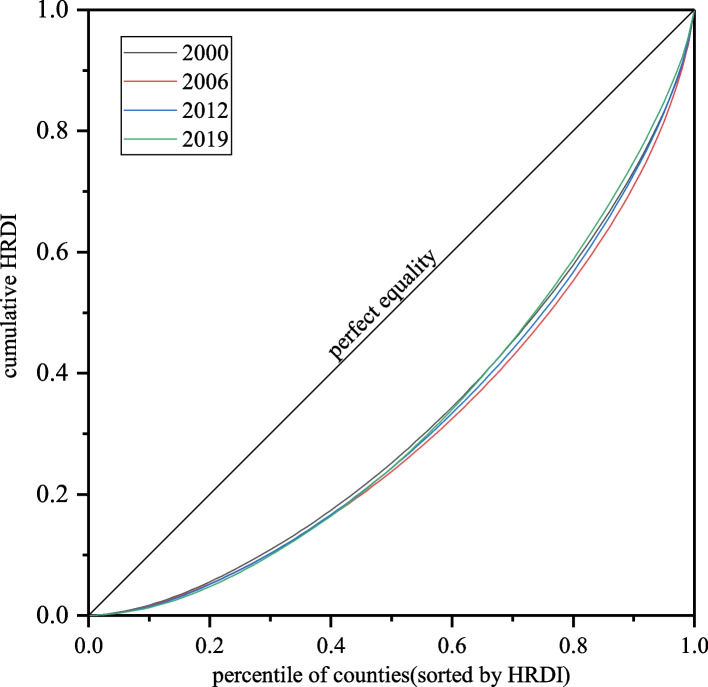
Lorenz curves of the HRDI among counties in China, 2000, 2006, 2012, and 2019. Note: The figure was generated using Origin software based on hospital beds distribution data

Due to the differences in the growth rate of the HRDI, the regional disparity changed. Figure 5 presents the allocation of the HRDI using Lorenz curves. From 2000 to 2006, the Lorenz curve shifted down and to the right, away from the 45° line, indicating that the disparity grew during that period. Slight improvements occurred during the 2006–2012 and 2012–2019 periods as the curves moved closer to the 45° line. More complicatedly, to the left of the 67th percentile of counties, the Lorenz curve for 2006, is above that for 2019. Then, these two curves intersected, as a result, a comparison of the disparities in these two years is graphically ambiguous [46]. Therefore, we needed to calculate the Gini coefficients to obtain accurate conclusions.

As shown in Table 3, at the national level, the overall Gini coefficient increased from 0.365 in 2000 to a peak of 0.391 in 2006; then, it decreased to 0.367 in 2019. The values and trends showed that there were large disparities in the HRDI among counties in China. Although a significant improvement occurred during the 2006–2019 subperiod, generally, the Gini coefficient did not decrease significantly during the entire period from 2000 to 2019.

**Table 3:** Gini coefficient and its decomposition results of the HRDI of counties in China, 2000–2019

Year	Total Gini Coefficient	Intraregional difference			Interregional difference			Contributions (%)		
		**East**	**Central**	**West**	**East-Central**	**East–West**	**Central-West**	G_***w***_	G_***nb***_	G_***t***_
2000	0.365	0.259	0.268	0.437	0.279	0.441	0.402	30.10	40.71	29.19
2001	0.371	0.264	0.267	0.453	0.281	0.448	0.409	30.29	39.44	30.27
2002	0.375	0.272	0.268	0.454	0.287	0.451	0.408	30.49	38.77	30.73
2003	0.375	0.275	0.268	0.450	0.293	0.453	0.403	30.36	39.55	30.09
2004	0.383	0.293	0.276	0.453	0.304	0.459	0.408	30.50	39.43	30.08
2005	0.385	0.305	0.279	0.444	0.317	0.463	0.401	30.28	41.22	28.50
2006	0.391	0.318	0.283	0.448	0.327	0.468	0.403	30.35	41.06	28.59
2007	0.389	0.310	0.290	0.445	0.326	0.462	0.402	30.54	40.26	29.20
2008	0.383	0.303	0.283	0.444	0.320	0.456	0.396	30.59	40.46	28.95
2009	0.381	0.303	0.284	0.446	0.320	0.449	0.393	31.00	38.85	30.15
2010	0.381	0.302	0.283	0.448	0.320	0.449	0.393	31.00	38.55	30.45
2011	0.379	0.300	0.279	0.445	0.317	0.447	0.390	31.00	38.63	30.37
2012	0.378	0.296	0.277	0.455	0.310	0.442	0.392	31.54	35.78	32.68
2013	0.379	0.293	0.280	0.462	0.305	0.438	0.397	31.88	34.43	33.69
2014	0.373	0.287	0.282	0.457	0.298	0.427	0.396	32.19	32.36	35.46
2015	0.373	0.280	0.290	0.458	0.296	0.424	0.400	32.22	32.31	35.47
2016	0.368	0.275	0.285	0.452	0.292	0.418	0.392	32.27	32.49	35.24
2017	0.370	0.275	0.286	0.455	0.294	0.422	0.395	32.14	33.20	34.66
2018	0.368	0.270	0.283	0.457	0.288	0.419	0.396	32.20	32.90	34.90
2019	0.367	0.212	0.274	0.489	0.266	0.421	0.409	32.27	29.79	37.95

The regional differences in the HRDI of the western region were the highest in each year, followed by those in the eastern region during the 2002–2014 period, and those in the central region in other years. The Gini coefficient for the central region changed gently with a fluctuation between 0.2674 and 0.2904, presenting an “M shape” with two peaks of 0.2901 and 0.2904 in 2007 and 2015, respectively, remaining almost stable during the period of 2000–2003 with a fluctuation range of only 0.003, and increasing during the 2000–2007 and 2012–2015 periods. The Gini coefficient for the eastern region showed an obvious downward trend with the largest fluctuation range being between 0.212 and 0.318, rising from 0.259 in 2000 to the peak of 0.318 in 2006, and then decreasing. The Gini coefficient for the western region showed a “wave-like” pattern with repeated fluctuations, but it generally increased by 0.052.

Moreover, it is essential to emphasize that intraregional factors exist not only within the eastern, central, and western regions but also within individual provinces. For instance, in 2020, the top five provinces with the highest Gini coefficients were Xinjiang, Inner Mongolia, Qinghai, Gansu, and Sichuan, with Gini coefficients of 0.5515, 0.5062, 0.4440, 0.4225, and 0.4214, respectively. These figures indicate a notable level of disparity in the allocation of hospital beds within these provinces.

The overall differences in the HRDI of counties between the three subregions fluctuated downward, with the largest difference being found between the eastern and western regions and the smallest disparity being found between the eastern and central regions. These results suggest that the differences between the western region and the eastern and central regions are major factors in the overall disparity in the HRDI of counties in China. Specifically, the difference between the western and eastern regions fluctuated, with a low of 0.418 in 2016 and a peak of 0.468 in 2006, showing an inverted “U-shaped” trend. Slightly lower than that between the western and eastern regions, the differences between the western and central regions generally displayed a “U shape”, although fluctuating repeatedly in some subperiods, and the Gini coefficient for 2019 (0.409) was slightly higher than that for 2000 (0.402). In contrast, the difference between the eastern and central regions changed gradually from 0.279 to 0.266, dividing the whole period into an increasing subperiod from 2000 to 2006 and a declining subperiod until 2019.

In terms of the contribution rate, in 2014 and before,  *G*_*nb*_, the interregional difference, was the primary contributing factor, responsible for approximately 40% of the overall differences. From 2015, *G*_t_, the trans-variation intensity, became the largest contributing component, accounting for approximately 35%. Compared with 2000, the percentage contribution of *G*_*w*_, the intraregional difference, was relatively stable, with a fluctuation between 30.10% and 32.27%; *G*_t_ increased from 29.19% to 37.95%, while  *G*_*nb*_ decreased from 40.71% to 29.79% and became the lowest contributing component in 2019. 

## Discussion

From 2000 to 2019, the number of hospital beds both per thousand people and per 100 square kilometers at the county level in China increased, but the growth rates varied across regions. The interpretation is that during this period, China achieved great economic development, and the government’s expenditure on healthcare increased dramatically [47–49]. However, there are large disparities in the dispersal of public revenue and the expenditure of county-level governments [50, 51], which represent the main body for health expenditures in China [52], causing a high degree of vertical imbalance in terms of the government’s fiscal capacity for the public healthcare system.

When measuring the level of hospital bed allocation using the HRDI, the eastern region was the highest, and the western region was the lowest. During the period studied, the HRDI of hospital beds in the western region increased faster than that in the eastern and central regions, while there was not much difference in the growth rate between the eastern and central regions. Within the western region, the growth rate of the HRDI of hospital beds in counties at the lower end of the scale was lower than that of counties at the higher end of the distribution. In contrast, within the eastern and central regions and at the national level, the growth rates of different counties fluctuated only in relatively small amplitudes. The proportion of counties with a lower HRDI declined, and the proportion of counties with a higher HRDI increased. The reason is that during the period, China increased both its total and per capita public health expenditure and, in particular, tilted public health expenditure toward underdeveloped areas such as the central and western regions [53, 54].

Going forward, by calculating the Dagum Gini coefficient, we found that there were large disparities in the HRDI. This finding is consistent with theliterature [33, 55]. Nonetheless, China has commenced a series of supporting policies and programs to coordinate regional development, directly suppressing the widening trend of the regional gap [56, 57]. Moreover, fiscal transfer payments redistributed funds to underdeveloped regions and corrected the imbalanced distribution of public health services [58, 59]. Thus, the overall disparity increased only slightly or just fluctuated.

Interregional disparity, the leading contributing factor in 2000, made the lowest contribution to the overall disparity in 2019. However, the contribution rate of intraregional disparity remained stable and contributed more than interregional disparity. This finding is consistent with the conclusion based on decomposing the regional differences in the health status of Chinese residents documented by Zhao, Wang [60]. The interpretation is that, in China, there are still enormous disparities within each subregion or even within a province [61]. For example, Guangdong is the richest province in China, but the economic development in northwest Guangdong is far behind that in the Pearl River Delta [62, 63], as are the basic public services, including health resources [64].

It is important to note that this paper primarily focuses on counties while excluding municipal districts, taking into consideration the differences in administrative power, public service provision (including hospital beds), and geographical characteristics between these two types of administrative regions. As a result, the findings above are applicable solely to counties in China. Despite this limitation, the study still holds value, as it provides significant insights into the allocation of hospital beds specifically at the county level.

The level of hospital beds serves as a critical indicator for estimating the healthcare service capacity of a region, as it reflects the allocation of other health resources, such as human resources and medical equipment [65]. Consequently, disparities in hospital bed availability can lead to inequalities in other health resources, such as physicians, nurses, and medical equipment, ultimately resulting in disparities in overall healthcare services [66–68]. In turn, these disparities can lead to health gaps among residents within a particular region [69, 70].

In light of the 'Healthy China 2030' plan, which aims to reduce the gaps in basic health services and health status between populations in different regions, it is essential for the government to address the significant disparities in hospital beds in counties, where approximately three-quarters of the total population resides. First and foremost, the government should increase its healthcare expenditure in regions with lower levels of bed resources, by enhancing local government finances via transfer payments or by using internal government assessments to incentivize local governments to expand the hospital bed supply. Doing so will help reduce the regional disparities in hospital beds and promote health equity. Given the significant intraregional disparities, the government should implement measures aimed at narrowing the disparities in hospital beds within the eastern, western, and central regions, and even within individual provinces. Second, efforts should be made to improve the efficiency of hospital bed utilization in areas with a low allocation of hospital beds by improving medical technology. Third, the government should encourage the establishment of healthcare communities between regions and refine the cross-regional healthcare insurance system to facilitate the sharing of hospital bed resources among different regions. Doing so will ensure that residents in areas with fewer hospital beds can also access essential healthcare services.

However, it is essential to acknowledge that achieving absolute equality, such as completely eradicating the regional disparities in the hospital bed supply, is an unattainable goal [71] due to the differences in regional economic development, population structure, health status, and medical requirements. Thus, the government should adopt a demand-oriented approach that not only considers the per capita level of hospital beds but also takes into account regional variations in the population structure and disease patterns. This approach involves assessing the specific demand for hospital beds based on these factors to determine the appropriate supply scale..

Meanwhile, our study has some limitations. First, health resources consist of a variety of factors, such as the health workforce, facilities, institutions, and beds. However, for the period from 2000 to 2020, data for other healthcare indicators in all counties were not available, except for hospital beds. Consequently, this study has chosen to focus solely on hospital beds as the representative indicator. While this choice can partially reflect the level of health resource allocation in counties, it does not provide a comprehensive representation. As a result, the findings of this study cannot fully reflect the overall disparity in health resource allocation in China.. Second, due to data limitations, we used the entire geographical area of a county when calculating the HRDI. This method may underestimate the level of hospital beds in counties with inaccessible areas and affect the estimation of regional disparities. Third, this paper estimated the disparity in hospital beds at the county level in China; however, it did not analyze the factors influencing the distribution pattern, which thus limits the policy implications that we can propose.

## Conclusions

This paper conducted a measurement and an analysis of the level of and changes in hospital beds across Chinese counties from 2000 to 2019, utilizing the health resource density index (HRDI) to estimate and decompose disparities and their variations. The study findings demonstrated a significant increase in the availability of hospital beds across Chinese counties during the study period. Nonetheless, substantial regional disparities continue to persist, showing an upward trend. These disparities hinder equitable access to healthcare services and the achievement of health equity. Our study emphasizes the importance of narrowing the disparities between counties to achieve the objectives outlined in the "Healthy China 2030" plan. The findings presented in this study contribute insights into the distribution of health resources at the county level, although, due to data constraints, we were limited to using hospital beds as the sole indicator for assessment.

## Data Availability

The datasets analyzed during the current study are available from the Research Platform of Big Data on China's Economy and Society, owned by CNKI, which is not publicly available. To request the data from this study, please contact the corresponding author Dr. Zuobao Wang (wzb812@163.com).
